# Stress affects the prediction of others’ behavior

**DOI:** 10.1371/journal.pone.0283782

**Published:** 2023-04-13

**Authors:** Sarah Witt, Sabine Seehagen, Norbert Zmyj

**Affiliations:** 1 Institute of Psychology, TU Dortmund University, Dortmund, Germany; 2 Faculty of Psychology, Ruhr University Bochum, Bochum, Germany; CHUV: Centre Hospitalier Universitaire Vaudois, SWITZERLAND

## Abstract

Predicting behavior of other people is vital for successful social interactions. We tested whether a stress-induced cortisol increase affects healthy young men’s prediction of another individual’s behavior. Forty-two participants were randomly assigned to a stress or to a control condition. Afterwards, they participated in a modified false-belief task that not only tests false-belief understanding but also the tendency to predict another person’s future behavior based on his former behavior. Subjective ratings and salivary cortisol concentrations revealed a successful stress induction. Stress did not affect participants’ attribution of false beliefs but it increased the probability to predict that a protagonist would act according to his former behavior. Recognizing that stress fosters the interpretation of others’ behavior following their former behavior and not their current goals extends previous research showing that stress fosters our own habitual behavior.

## Introduction

To form and maintain social relationships, we frequently rely on social cognitive skills. But how do these skills work under stressful circumstances? In the present study we examined whether stress-induced cortisol elevations affect participants’ performance in a false-belief task.

Stress arises when individuals perceive a threat to their physiological or psychological well-being [1]. A large body of literature demonstrates that stress affects our thoughts and feelings through a series of physiological processes and hormonal changes. Controlled laboratory experiments revealed that stress, via glucocorticoid hormones, affects cognitive functions like memory (for a review, see [2]), learning [3], and decision making [4]. However, while a large body of research on stress effects on memory and learning exists, comparatively few studies focused on social cognition. This is surprising since social cognitive skills may help to succeed in social interactions. Theory of Mind, the ability to attribute mental states to oneself and others [5], is one of the most widely studied social cognitive skills. A functional Theory of Mind enables humans to explain and predict others’ behavior, thereby maintaining social relationships. However, until now, we do not know exactly how Theory-of-Mind competencies work under stressful circumstances.

A few studies investigated the effect of stress on social cognition so far. One study [6] used the Reading the Mind in the Eyes Test–Revised (RMET–R, [7]) and the Movie for the Assessment of Social Cognition (MASC; [8]) to assess social cognition in a stress condition and a control condition. Neither RMET–R scores nor MASC scores revealed main effects of stress. However, the MASC scores revealed an interaction effect between stress and sex. While the correlation between cortisol responses and MASC scores was positive among men, it was negative among women. Thus, the association of cortisol with mental state reasoning was sex specific [6]. Sex differences were also found for the effect of stress on perspective taking. Women’s perspective taking was faster in the stress condition compared to the control condition. However, men in the stress condition were slower compared to the control condition [9]. A placebo-controlled study revealed that increases in cortisol concentrations per se were not associated with emotion recognition, but interacted with variables like sex, emotion, and task difficulty [10].

According to previous stress research, there are several reasons to assume that stress affects Theory of Mind. First, there is a very high density of glucocorticoid and mineralocorticoid receptors in the prefrontal cortex (PFC) indicating that this area is highly stress sensitive [11,12]. In line with these findings, there is evidence that some cognitive functions mediated by the PFC are affected by high glucocorticoid levels after stress [13,14]. Furthermore, the PFC is known for its important role in social aspects of cognition [15,16]. Taken together, the stress-induced reduction of PFC capacity might affect social reasoning.

Second, coping with stressful events includes a shift towards less demanding cognitive processes (for a review, see [17]). For example, stress favored less demanding strategies in a spatial learning task [18] and an instrumental learning task [19]. These studies revealed that stressed participants used less demanding strategies like stimulus-response learning or habitual behavior compared to control participants who used more demanding strategies like spatial learning or goal-directed behavior.

A stress-related shift towards less (PFC-) demanding processes may also occur with regard to social cognition. False-belief tasks are frequently used as a measure of Theory-of-Mind performance [20]. It is necessary to suppress one’s own knowledge to correctly infer the protagonist’s knowledge. Therefore, reasoning about others’ mental states in this scenario is likely more resource demanding than relying on one’s own mental states and requires inhibitory control. In an adapted version of a false-belief task, even adults were biased by their own knowledge when reasoning about someone else’s mental states [21]. In this study, participants observed a protagonist placing a violin in one of four containers. The violin was transferred to another container in his absence and the containers switched locations afterwards. Participants were asked to predict where the protagonist would look for the violin on his return. It was manipulated which container the violin was transferred to and whether the participants were informed about this outcome. Participants were particularly likely to attribute their own knowledge about the violin’s location to the protagonist when there was a plausible explanation for the protagonist not to act according to his false belief about the violin’s location. For example, when the violin was in the container that occupied the location where the violin had originally been participants were particularly cursed by their own knowledge. Put differently, adults did not consistently predict others to perform goal-directed behavior (i.e., looking for an object in a container where the other person thought it was) but instead sometimes predicted others’ actions on the basis of their former behavior (i.e., returning to the location where the other person had gone to in the past). The likelihood of using such a cognitive shortcut might increase under stress.

To examine whether a stress-induced cortisol increase affects the prediction of others’ behavior, we exposed participants to an acute stressor or a control condition prior to conducting a false-belief procedure based on Birch and Bloom’s task [21]. We hypothesized that stressed participants would show an increased tendency to predict another person’s future behavior being based on their own previous behavior. Specifically, we predicted that compared to their non-stressed counterparts, stressed participants would be more likely to assume the protagonist’s search to be guided by location (i.e., considerations where he stored the violin before) rather than container identity.

We also hypothesized that stressed participants would be biased by their own knowledge in a false-belief task more often than control participants. That is, they would be more likely to predict that the protagonist would look for the violin at its current location than participants in the control condition.

## Materials and methods

### Participants and design

Forty-two healthy men between 19 and 33 years of age (*M* = 24.14, *SD* = 3.52) and with a body-mass-index between 18 and 28 kg/m^2^ participated in this study. We used a between-subjects design in which participants were randomly assigned to either a control condition (*n* = 21) or a stress condition (*n* = 21). Four additional participants from the stress condition were excluded from further analysis. They took their hand out of the water between 50 and 95 seconds after the beginning of the stress induction. Although they remained in the social stress situation (e.g., evaluation by the experimenter, video recording), their mean cortisol increase from baseline to 20 minutes after SECPT onset (*M* = 0.32 nmol/l, *SD* = 0.82) was lower compared to the cortisol response of participants from the stress condition who kept their hand in the water for the entire time span (*M* = 6.61 nmol/l, *SD* = 1.18; *t*(23) = 2.28, *p* = .032).

Participants from the stress and control condition did not differ in age (*M*_control_ = 25 years; *SD*_control_ = 4.01; *M*_stress =_ 23 years, *SD*_Stress_ = 2.71; *t*(40) = 1.80, *p* = .079), BMI (*M*_control_ = 23.4 kg/m^2^, *SD*_control_ = 2.27; *M*_stress_ = 23.9 kg/m^2^, *SD*_stress_ = 2.65; *t*(40) = -0.70, *p* = .489) or the highest level of education (*U* = 152.00, *p* = .069).

Factors like alcohol [22], nicotine [23], exercising [24], or food intake [25] contribute to the fluctuation of cortisol levels. Moreover, several studies reported altered cortisol stress reactivity in clinical samples (for a review, see [26]). Therefore, we used screening questionnaires to check exclusion criteria like regular smoking, past or current psychiatric, neurological or endocrine disorders, and medication intake. Furthermore, participants were instructed not to do physical exercises or drink caffeine or alcohol on the day of their participation. Moreover, they were asked not to eat or drink anything except water within 90 minutes prior to the experiment. All participants provided written informed consent and received a monetary compensation for participation of 10 €. The experiment was approved by the ethics commission of TU Dortmund University.

### Procedure

To control for the circadian rhythm of cortisol secretion all sessions took place in the afternoon. Participants underwent either the socially evaluated cold pressor test (SECPT; [27]) or a non-stressful control procedure. During the SECPT participants had to immerse their right hand into ice water (0–2°C). Within this three-minute period, participants were video-recorded and monitored by a reserved female experimenter. In the control condition, participants immersed their right hand into warm water (35–37°C). Within this three-minute period, they were neither video-recorded nor observed by an experimenter. Immediately after the SECPT or control procedure, participants used a scale ranging from 0 (‘‘not at all”) to 100 (‘‘very much”) to rate how difficult it was to keep their hand immersed, and how unpleasant, stressful, and painful they experienced the treatment.

**Fig 1:**
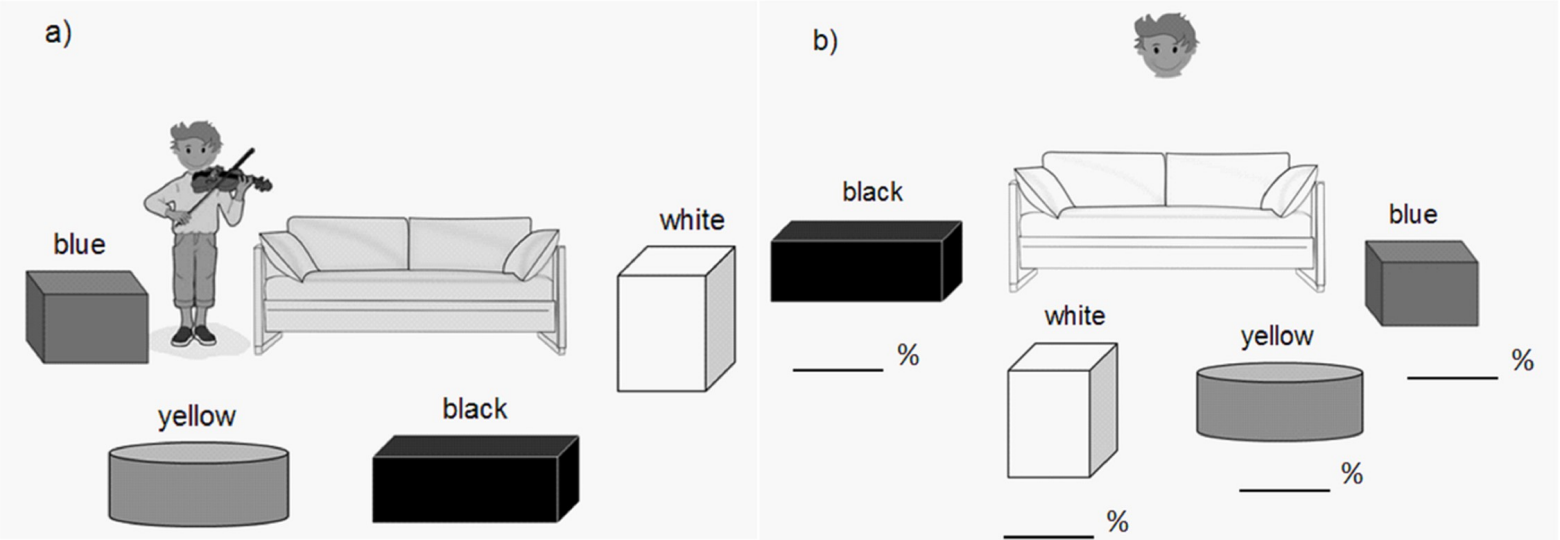
The first and last picture of the false-belief task. Pictures of the false-belief task show the set-up of the containers before the protagonist left the room (a) and after his return (b).

Twenty minutes after the stress or control manipulation, participants completed a false-belief task (adapted from [21]). The participants listened to a story told by a second female experimenter while pictures were presented to illustrate the storyline. On the first picture there was a boy standing in a room and holding a violin. Four containers (blue, yellow, black, and white) were distributed around a sofa in the middle of the room (Fig 1A). Participants were told that the boy called Niklas finished playing his violin and put it in the blue container. The second picture showed Niklas leaving the room to play outside. The third picture depicted another boy in the room who put the violin in the yellow container. The participants were told that this was Niklas’ brother transferring the violin from the blue to the yellow container while Niklas was still playing outside. At this point, two control questions were asked to ensure that the participants correctly remembered the different positions of the violin throughout the story: “Where did Niklas put the violin?” and “Where did his brother put the violin?”. The fourth picture showed the brother relocating the containers. The next picture showed the final set-up of the containers (Fig 1B). The violin was neither in the blue container where Niklas put it, nor in the location where the blue container was placed originally. Finally, the participants were asked to rate the probability of Niklas looking for his violin in each of the containers first, distributing a total of 100% between four containers.

To assess salivary cortisol concentrations, three saliva samples were collected throughout the experiment. Salivettes (Sarstedt, Nümbrecht, Germany) were used to take samples before (baseline), as well as 20 and 35 minutes after the onset of the treatment. Samples were stored at -20°C until biochemical analysis. The fraction of free cortisol was assessed using an immunoassay (IBL, Hamburg). Mean intra- and inter-assay coefficients of variance were below 9%.

Statistical analyses were conducted using IBM SPSS Statistics (Version 29.0) and JASP (Version 0.16.4). T-tests for independent samples were used to compare the mean subjective stress ratings of both conditions. A repeated-measures ANOVA was conducted to check if there were differences in salivary cortisol concentrations between both conditions throughout the experiment. To test the main hypotheses, t-tests for independent samples were used to check if there were differences in the participants’ mean probability ratings for each of the containers.

## Results

### Stress induction

Analyses of subjective stress ratings and salivary cortisol concentrations revealed a successful stress induction. The SECPT was rated as more unpleasant (*t*(30.98) = -6.30, *p* < .001, *d* = 1.94), more stressful (*t*(27.07) = -5.84, *p* < .001, *d* = 1.80), and more painful (*t*(30.36) = -10.93, *p* < .001, *d* = 3.38) than the control condition. Furthermore, participants from the stress condition found it more difficult to keep their hand immersed in the water compared to participants from the control condition (*t*(30.41) = -6.71, *p* < .001, *d* = 2.07; see Table 1 for descriptive data).

**Table 1:** Mean (SD) subjective ratings and salivary cortisol in the stress and control condition.

	control	stress
subjective ratings		
difficult	4.76 (15.37)	52.86 (29.01)
unpleasant	7.62 (14.46)	49.05 (26.44)
stressful	4.29 (10.28)	37.62 (24.06)
painful	2.38 (10.91)	58.10 (20.64)
salivary cortisol (nmol/l)		
baseline	5.57 (2.72)	5.61 (3.97)
+20 minutes	7.49 (5.04)	12.22 (5.78)
+35 minutes	6.78 (5.27)	9.65 (3.64)

**Fig 2:**
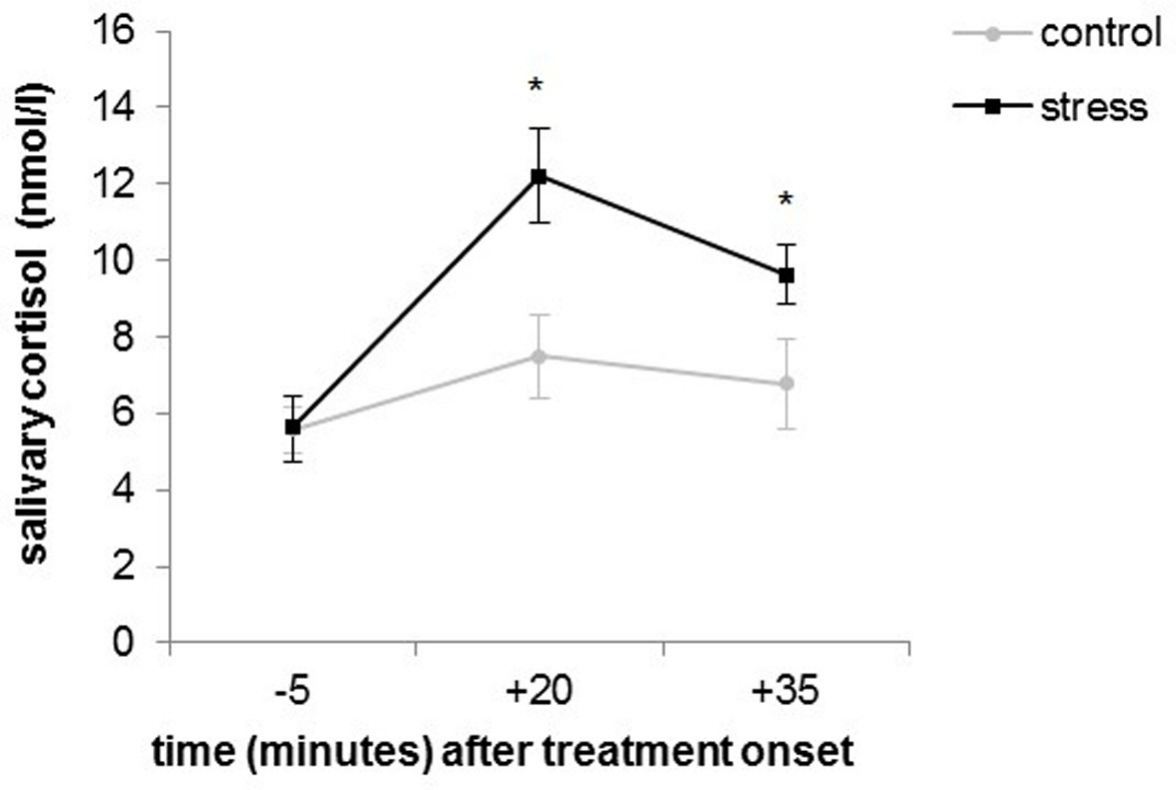
Mean salivary cortisol concentrations of participants from the control and stress condition throughout the experiment. Error bars represent standard errors.

Due to skewness, cortisol data were log-transformed for use in further analyses. A repeated-measures ANOVA conducted on salivary cortisol revealed significant main effects of condition (*F*(1, 39) = 4.47, *p* = .041, *η*_*p*_^*2*^ = .10) and time (*F*(1.33, 51.79) = 33.04, *p* < .001, *η*_*p*_^*2*^ = .46), as well as a significant condition × time interaction (*F*(1.33, 51.79) = 11.94, *p* < .001, *η*_*p*_^*2*^ = .23). Fig 2 shows that conditions differed in cortisol concentrations 20 minutes (*t*(40) = -3.08, *p* = .004, *d* = -0.95) and 35 minutes (*t*(39) = -2.77, *p* = .009, *d* = -0.86) after the SECPT or the control procedure, respectively. However, baseline cortisol levels did not differ between conditions (*t*(40) = -0.24, *p* = .815, *d* = -0.07). This indicates that the SECPT induced a physiological stress response, which was present throughout task performance.

### False-belief task

**Fig 3:**
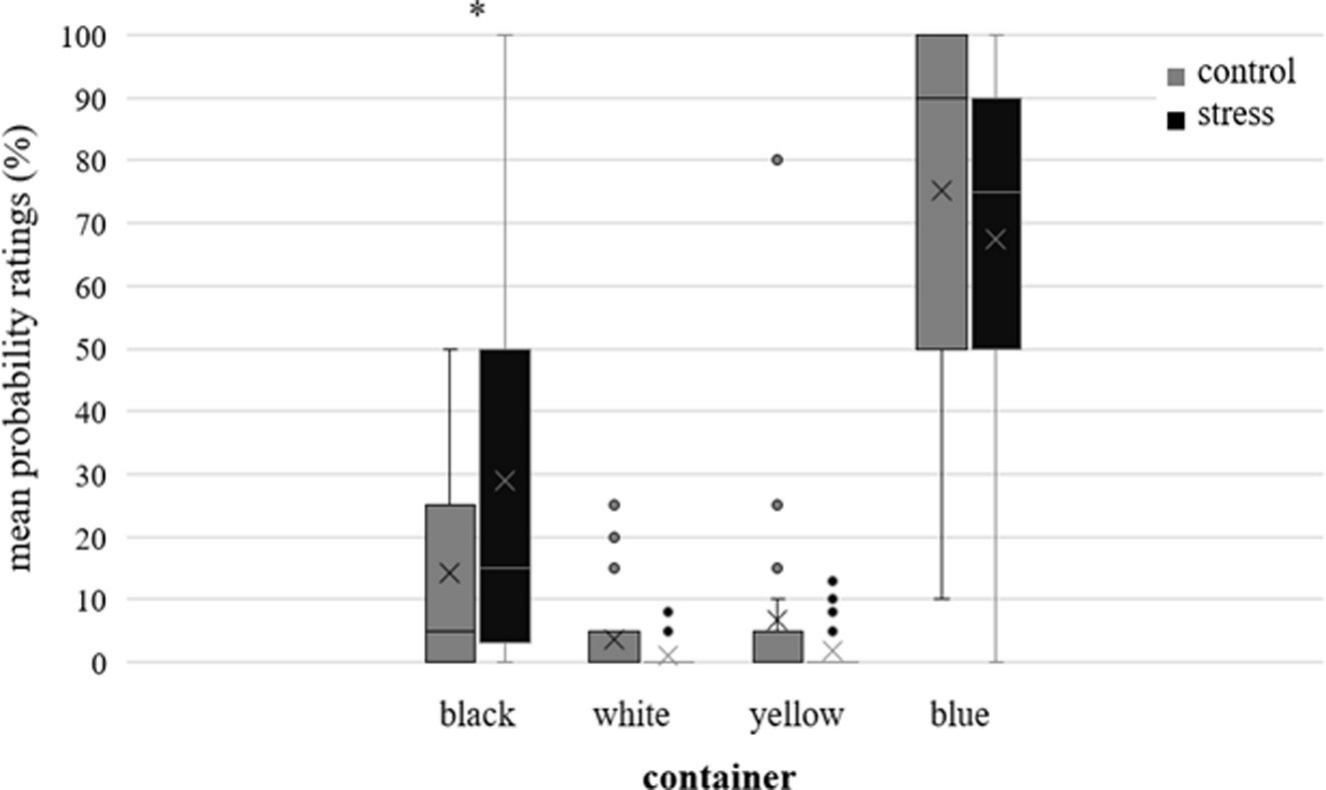
Boxplots representing the ratings of the probability that Niklas would look into the black, white, yellow, or blue container first. The lower and upper box boundaries represent the lower and upper quartiles, respectively. The horizontal line inside the box represents the median. When no horizontal line is present inside a box, the median is 0. The cross represents the mean. The lower and upper whiskers represent minimum and maximum scores, respectively. Dots outside the box represent values that deviate from the upper quartile by more than 1.5 times the interquartile range.

Mean probability ratings for the containers from the false-belief task are shown in Fig 3. The black container occupied the former location of the blue container in which Niklas deposited the violin. To test the hypothesis that stress increases the likelihood of predicting behavior based on a person’s former actions, we analyzed how likely participants thought it was for Niklas to look in the black container first. Participants in the stress condition rated the probability for Niklas to look into the black container first significantly higher than participants from the control condition (*t*(30.58) = -1.84, *p* = .038 (one-sided), *d* = -0.567). Bayesian independent *t*-tests confirmed the results (BF_-0_ = 2.165; error % < 0.0001), indicating that the data are 2.165 times more likely to occur under the directional alternative hypothesis compared to the null hypothesis. To test the hypothesis that stress influences the ability to suppress one’s own knowledge and to correctly attribute a false belief to another person, we analyzed the likelihood that participants ascribed to Niklas looking in the blue container first. The participants’ ratings of the probability that Niklas would look into the blue container first did not differ between conditions (*t*(40) = 0.81, *p* = .212 (one-sided), *d* = 0.25; BF_+0_ = 0.601; error % < 0.00001). We did not have any assumptions concerning the yellow and the white container. Nevertheless, we conducted *t*-tests to check if there were unexpected differences between both conditions. The probability ratings for the yellow container, which included the violin at the end of the story did not differ between conditions (*t*(21.84) = 1.22, *p* = .237, *d* = 0.375; BF_10_ = 0.544; error % = 0.006). The ratings of the probability that Niklas would look into the white container first did not differ between conditions, (*t*(24.48) = 1.57, *p* = .131, *d* = 0.48; BF_10_ = 0.809, error % = 0.006) either.

## Discussion

The present study investigated the effect of acute stress on the performance in a false-belief task. The SECPT resulted in a cortisol increase compared to the non-stressful control procedure. Participants from both conditions did not differ in their tendency to attribute their own knowledge to the protagonist. Thus, stressed participants were not cursed any more by their own knowledge when reasoning about the protagonist’s false belief than were their non-stressed counterparts. However, stressed participants did show a stronger tendency to assume that the protagonist’s future behavior would be a repetition of his former behavior compared to non-stressed participants. Specifically, compared to the control condition, participants in the stress condition rated it more likely that the protagonist would look for his object at the original location. This finding ties in with studies suggesting that stress causes a shift from goal-directed to habitual behavior [18,19]. The present study shows that a shift from goal-directed behavior to actions based on former behavior does not only occur for a person’s own behavior but also when reasoning about others’ behavior. However, it should be noted that the present study refers to one-time behavior shown in the past, while others referred to behavior repeated throughout several trials [19]. Therefore, future studies should examine how stress affects the prediction of others’ habitual behavior.

Habitual behavior is less resource demanding and allows fast reactions which are often needed in stressful situations [17]. Regarding the false-belief task, it might be efficient to take the former location of the relevant container into consideration. Since the protagonist as well as the participants knew where this location was, deciding upon this option did not require to differentiate between their different states of knowledge. Furthermore, suppressing salient information (i.e., the original location of the violin) requires additional resources. Habitual behavior under stress allows to respond immediately and might be beneficial on the one hand. On the other hand, a lack of flexibility might have negative outcomes in social conflicts like rigid reliance on stereotypes and prejudice.

A recent study revealed that stressed participants were faster in the detection and extraction of probability-based regularities in a reaction time task compared to a control condition [28]. This result indicates that stress promotes some kind of regularity detection. Probably, the stressed participants in the present study also focused on the detection of regularities when they were asked to predict the participant’s behavior. This might explain why they rated the probability for the black container higher than participants from the control condition. The black container was placed at a location the protagonist previously went to. Under stress, the probability of the protagonist doing so again might have appeared elevated.

In contrast to our second hypothesis, stress did not affect the attribution of a false belief. Participants in the stress condition did not show a stronger bias towards their own knowledge compared to participants in the control condition. This is surprising because of the high stress-sensitivity of the PFC [12] and its important role in false-belief attribution [15]. One way to explain these null results is that the task was possibly too easy for adults. However, previous studies using more sophisticated mindreading tasks did not provide straightforward effects of stress on social cognition [6]. Stressed adults might have enough cognitive resources available to infer false beliefs correctly when they are explicitly asked to do so. This might change in the context of implicit Theory-of- Mind tasks in which processing a protagonist’s belief is not essential to answering the test questions (e.g., [29]). Another explanation of the null results is that we did not measure potential mediators such as executive functions. Executive functions like inhibitory control are mainly controlled by prefrontal regions of the brain. Moreover, they are essential for solving false-belief tasks. However, previous studies on the effect of stress on inhibition revealed inconsistent findings. While some studies indicated a stress-induced impairment of inhibitory control [14,30], others revealed that stress [31] or cortisol administration [32] enhances inhibition. Future research should address the question if more implicit Theory-of-Mind tasks are affected by stress and if or how executive functions mediate effects of stress on social cognition.

Salivary cortisol concentrations are influenced by menstrual cycle phases or the use of oral contraceptives [33]. To eliminate this confounding variable, the sample of the present study was restricted to male participants. Future studies should involve female subjects while systematically controlling for the use of oral contraceptives and cycle phases on the day of participation. Previous studies investigating mixed samples found sex differences in mental state reasoning [6] as well as self-other distinction [9]. However, results of these studies were contradictory. In [6] stress enhanced mental state reasoning in men and reduced it in women. In [9] stress reduced self-other distinction in men and enhanced it in women. Since self-other distinction is a prerequisite for mental state reasoning, further research is needed to clarify these opposite effects.

Laboratory stressors typically induce acute and temporary responses. Ethical considerations do not allow inducing chronic stress. However, questionnaires or interviews could be used to investigate the association between chronical stress and social cognition. Chronical stress is associated with structural changes in the PFC [34]. Therefore, it should be clarified if people who consistently suffer from stress show deficits in social cognition. In the view of test theory, using a one-trial task to investigate the effect of stress on social cognitive skills might be a shortcoming of the present study. However, there are several studies in adult Theory of Mind research that used single trial false-belief tasks as well [35–37]. Two of them even used adapted versions of the same task as we used in the present study [21] and revealed that participants’ mood [35] as well as their mind-set [37] affected if the prediction of others’ behavior was biased by their own knowledge. Moreover, using a single trial task prevents carry-over effects. Our results indicate that stress modifies answers in a certain Theory-of-Mind task. Future studies should prove if this effect applies to other tasks as well. In sum, stress did not affect participants‘ attributions of false beliefs but it increased the probability to predict others’ behavior by referring to their former actions. Previous research focused on the influence of stress on the own habitual behavior. Addressing the influence of stress on the interpretation of others’ behavior as habitual might be a promising avenue for further research in social cognition and social relationships.

## Supporting information

S1 Dataset The dataset used for the analyses reported in the manuscript. (XLS)
